# Anaesthetists' and surgeons' attitudes towards informed consent in the UK: an observational study

**DOI:** 10.1186/1472-6939-11-2

**Published:** 2010-02-23

**Authors:** AAB Jamjoom, S White, SM Walton, JG Hardman, IK Moppett

**Affiliations:** 1 Nottingham University Medical School, Queen's Medical Centre, Nottingham, NG7 2UH, UK; 2 University Department of Anaesthesia, Queen's Medical Centre, Nottingham, NG7 2UH, UK; 3 Department of Anaesthesia, Royal Sussex County Hospital, Brighton, BN2 5BE, UK; 4 Department of Anaesthesia, Eastbourne District General Hospital, Eastbourne, BN21 2UD, UK

## Abstract

**Background:**

The attitudes of patients' to consent have changed over the years, but there has been little systematic study of the attitudes of anaesthetists and surgeons in this process. We aimed to describe observations made on the attitudes of medical professionals working in the UK to issues surrounding informed consent.

**Method:**

A questionnaire made up of 35 statements addressing the process of consent for anaesthesia and surgery was distributed to randomly selected anaesthetists and surgeons in Queen's Medical Centre (Nottingham), Royal Sussex County Hospital (Brighton) and Eastbourne District General Hospital (Eastbourne) during 2007. Participants were asked to what extent they agreed with statements regarding consent.

**Results:**

Of 234 questionnaires distributed, 63% were returned. Of the respondents 79% agreed that the main purpose of the consent process is to respect patient autonomy. While 55% of the examined cohort agreed that the consent process maybe inappropriate as patients do not usually remember all the information given to them. Furthermore, 84% of the participants agreed that what the procedure aims to achieve should be explained to the patient during the consent process. While of the participants, over 70% agreed that major risks of incidence greater than 1/100 should be disclosed to the patient as part of the consent process.

**Conclusions:**

The majority of respondents appear to hold attitudes in standing with current guidelines on informed consent however there was still a significant minority who held more paternalistic views to the consent process bringing into question the need for further training in the area.

## Background

Consent has become a topic of heightened interest and debate, not only within the medical profession but also in the wider public media. An increasing number of medico-legal cases and the introduction of national guidelines by the UK Department of Health have formalised the way consent is obtained and in particular how potential risks are disclosed [1]. Heightened awareness has led to a considerable amount of medical research into the consent process. Most of the published reports focused on patients' as opposed to doctors' attitudes towards consent [2-7]. A number of studies have looked at consent in a particular setting such as in intensive care [8], medical research [9], plastic surgery [10], interventional radiology [11] and paediatric surgery [12], or in managing a specific problem such as laparoscopic cholecystectomy [13] and emergency abdominal surgery [14].

Most of the studies that looked at doctors' attitudes towards consent were performed outside the UK and none of them looked at the opinions of doctors in relation to their specialty [15-18]. Surgeons have traditionally been more involved in the consent process than anaesthetists and anecdotally, surgeons appear to be more inclined to involve patients in decision making than anaesthetists. Therefore, the authors felt it was appropriate to study the attitudes of anaesthetists and surgeons towards the entire process of consent in the UK. Our aim was to assess whether surgeons' and anaesthetists' attitudes towards consent were in standing with current national guidelines.

## Methods

A questionnaire was distributed in Queen's Medical Centre in Nottingham, the Royal Sussex County Hospital in Brighton and Eastbourne District General Hospital in Eastbourne during 2007 to a randomly selected cohort of anonymous anaesthetists and surgeons. The cohort consisted of those surgeons and anaesthetists present during theatre lists in main theatre suites at the respective hospitals over a 2 week period. The questionnaire was made up of 35 statements that addressed issues concerning consent for anaesthesia and surgery [Additional File 1]. The participants were asked to what extent they agreed with statements about consent on a five point Likert scale [19]; 'five' indicated complete agreement with the statement and 'one' showed complete disagreement. The statements in the questionnaire were grouped as follows:

**1**. Eight statements regarding the main purposes of the consent process

**2**. Seven statements related to why consent may be unnecessary

**3**. Seven statements related to what should be explained during the consent process

**4**. Six statements related to the factors which affect the amount of information given to patients during the consent process

**5**. Seven statements related to the levels of major and minor risks that should be disclosed during the process of consent

Our primary hypothesis was that both surgeons and anaesthetists should agree with the following statements:

**1**. Respect for a patient's right to autonomy is one of the main purposes of the consent process

**2**. That consent process maybe inappropriate as most patients do not usually remember all the information given to them during the consent process

**3**. That what the procedure aims to achieve should be explained to the patient as part of the consent process

**4**. That the complexity of the procedure affects the amount of information conveyed to the patient during the consent process

The questions related to the primary hypothesis were identified as important themes from the consent guidelines. They were asked in the middle of the questionnaire, and were not identified as such, to avoid unintended bias in the answers.

The local ethics committee waived the requirement for formal ethical approval because our study comprised an anonymised questionnaire; individuals provided consent by completing the questionnaire.

Before use, the questionnaire was piloted with ten medical students. This pilot highlighted grammatical ambiguities that were corrected for the final version of the questionnaire. This was re-examined by the pilot cohort before distribution to the participants. Face-to-face interviews were conducted with the initial pilot cohort to check that written responses reflected the respondents meaning and intent; there were no discrepancies.

The intended participants in this survey were consultants, registrars and senior house officers (SHO) in anaesthesia and surgery, of both sexes and varying age and years of experience, who were working at the Queen's Medical Centre in Nottingham, the Royal Sussex County Hospital in Brighton and Eastbourne District General Hospital in Eastbourne during 2007.

### Statistical analysis

Analysis of data was undertaken using Statistical Package for Social Sciences, Version 15.0 (Chicago, Illinois, USA). Raw data was analysed by taking answers of 1 and 2 on the Likert scale to indicate disagreement with the given statement while answers of 4 and 5 to indicate agreement.

The reliability and validity of the questionnaire was assessed by calculating the correlation statistics for intra and inter questionnaire groups of questions. The inter-group correlation co-efficient was calculated by comparing the responses to the four questions of the primary hypothesis.

## Results

Of 234 questionnaires distributed, 148 were returned - a response rate of 63% and all the participants responded to all the statements in the questionnaire. The demographics of the respondents are detailed in Table 1. The respondents profile is similar to that reported by the Royal College of Anaesthetists census except for the proportion of trainees which was higher in this project [20]. The inter-group question correlation co-efficient was 0.6.

**Table 1:** Summary of participant demographics

	Portion percentage (n)	Consultant percentage (n)	Registrar percentage (n)	SHO percentage (n)
**Surgeons**	40 (59)	73 (43)	24 (14)	3 (2)
**Anaesthetists**	60 (89)	62 (55)	29 (26)	9 (8)

With regard to the primary questions [Table 2], 79% of participants agreed with the statement that the main purpose of the consent process is to respect patient autonomy. While 55% of the examined cohort agreed that the consent process maybe inappropriate as patients do not usually remember all the information given to them. Furthermore, 84% of the participants agreed that what the procedure aims to achieve should be explained to the patient during the consent process. Finally, 65% of the cohort agreed that the amount of information conveyed to the patient is affected by the complexity of the procedure.

**Table 2:** Participant response to the primary hypothesis questions

**No**.	Question	Percentage of participants who agreed (n)	Percentage of participants who disagreed (n)
**1**	Respect for a patient's right to autonomy is one of the main purposes of the consent process	79 (117)	7 (10)
**2**	That consent process maybe inappropriate as most patients do not usually remember all the information given to them during the consent process	55 (82)	26 (39)
**3**	That what the procedure aims to achieve should be explained to the patient as part of the consent process	84 (124)	6 (9)
**4**	That the complexity of the procedure affects the amount of information conveyed to the patient during the consent process	65 (96)	22 (33)

### 1. What are the main purposes of the consent process?

The main purposes of informed consent for anaesthetists were: informing about risks (85%) and respect for patient autonomy (75%). Smaller proportions viewed 'patient benefit' as a main purpose: improving the doctor-patient relationship (60%); improving patient compliance (40%) and reducing patient anxiety (57%). This correlated closely to the attitudes to surgeons however higher percentages agreed with these statements compared to the anaesthetists [Table 3]. The intra-group question correlation co-efficient was 0.92.

**Table 3:** What are the main purposes of the consent process?

		Percentage of participants who agreed (n)		Percentage of participants who disagreed (n)	
No	Question	Surgery	Anaesthesia	Surgery	Anaesthesia
**1**	Inform the patient about risks and complications	97 (57)	85 (76)	0 (0)	6 (5)
**2**	Respect patient's right to autonomy	85 (50)	75 (67)	2 (1)	10 (9)
**3**	Educate the patient about alternative treatment options	80 (47)	65 (58)	8 (5)	11 (10)
**4**	Provide he doctor with greater protection from medical litigation	63 (37)	62 (55)	22 (13)	19 (17)
**5**	Inform the patient about the desired benefits of the procedure	95 (56)	70 (62)	2 (1)	16 (14)
**6**	Improve doctor-patient relationships	51 (30)	60 (53)	22 (13)	12 (11)
**7**	Improve patient compliance to their medical care	32 (19)	40 (36)	27 (16)	30 (27)
**8**	Reduce patient anxiety about the procedure	49 (29)	57 (51)	24 (14)	13(12)

### 2. Can the consent process be inappropriate or unnecessary?

A significant minority of anaesthetists felt that consent may be unnecessary or inappropriate because of concerns that: disclosing information about potentially harmful risks may be worrying and disadvantageous for the patient (22%); informing patients about details of alternative treatment modalities may be confusing (38%); and discussion of risks during informed consent may dissuade the patient from undergoing a procedure that may benefit him/her (30%). The results from surgeons were similar [Table 4]. The intra-group question correlation co-efficient was 0.82.

**Table 4:** Are there aspects of the consent process that are inappropriate or unnecessary?

		Percentage of participants who agreed (n)		Percentage of participants who disagreed (n)	
No	Question	Surgery	Anaesthesia	Surgery	Anaesthesia
**1**	Most patients trust their doctor to decide what is best for them	22 (13)	18 (16)	68 (40)	60 (53)
**2**	Most patients depend on their doctor to make the decision for them	19 (11)	19 (17)	61 (36)	52 (46)
**3**	Disclosing information about potentially harmful risks may be worrying and disadvantageous for the patient	22 (13)	22 (20)	66 (39)	55 (49)
**4**	Informing patients about details of alternative treatment modalities may be confusing	20 (12)	38 (34)	66 (39)	42 (37)
**5**	Discussion of risks during informed consent may dissuade the patient from undergoing a procedure that may benefit them	17 (10)	30 (27)	64 (38)	45 (40)
**6**	Most patients do not usually *understand *all the information given to them during the process of consent	36 (21)	37 (33)	46 (27)	38 (34)
**7**	Most patients do not usually *remember *all the information given to them during the process of consent	56 (33)	55 (49)	27 (16)	26 (23)

### 3. What should be explained to the patient during the process of consent?

Major topics of explanation highlighted by both surgeons and anaesthetists included: what the procedure entails, aims to achieve, realistic outcomes and the possibility of morbidity and mortality; however, a higher percentage of surgeons compared to anaesthetists agreed with all these statements [Table 5]. More anaesthetists than surgeons (7 vs 0%) disagreed with explaining any additional procedures that may be necessary. The intra-group question correlation co-efficient was 0.05.

**Table 5:** What should be explained to the patient during the process of consent?

		Percentage of participants who agree (n)		Percentage of participants who disagree (n)	
No	Question	Surgery	Anaesthesia	Surgery	Anaesthesia
**1**	What the procedure entails	92 (54)	83 (74)	2 (1)	4 (4)
**2**	What the procedure aims to achieve	97 (57)	75 (67)	0 (0)	10 (9)
**3**	Additional procedures that are likely to be necessary	95 (56)	79 (70)	0 (0)	8 (7)
**4**	A realistic outcome/results for the procedure	95 (56)	83 (74)	0 (0)	4 (4)
**5**	Alternative forms of treatment	75 (44)	85 (76)	10 (6)	3 (3)
**6**	The possibility of death (if present)	83 (49)	65 (58)	5 (3)	16 (14)
**7**	The possibility of significant disability (eg: stroke/paralysis)	86 (51)	70 (62)	7 (4)	16 (14)

### 3. What factors affect the amount of information conveyed to the patient during the consent process?

A high percentage of the participants felt that patient age, level of education, inquisitiveness and complexity of the procedure affected the amount of information conveyed to the patient [Table 6]. Of the anaesthetists, 71% and 73% agreed that patient's inquisitiveness and the complexity of the procedure affected the amount of information conveyed respectively. The intra-group question correlation co-efficient was 0.99.

**Table 6:** What factors affect the amount of information conveyed to the patient during the consent process?

		Percentage of participants who agree (n)		Percentage of participants who disagree (n)	
No	Question	Surgery	Anaesthesia	Surgery	Anaesthesia
**1**	Patient age	36 (21)	54 (48)	49 (29)	22 (20)
**2**	Patient's level of education	29 (17)	48 (43)	46 (27)	30 (27)
**3**	Patient's inquisitiveness	46 (27)	71 (63)	34 (20)	18 (16)
**4**	Complexity of procedure	53 (31)	73 (65)	34 (20)	15 (13)
**5**	How busy the doctor is at the time	8 (5)	24 (21)	73 (43)	49 (44)
**6**	Whether the patient is private or NHS	5 (3)	12 (11)	83 (49)	76 (68)

### 4. At what incidence should risk be disclosed?

**Figure 1:**
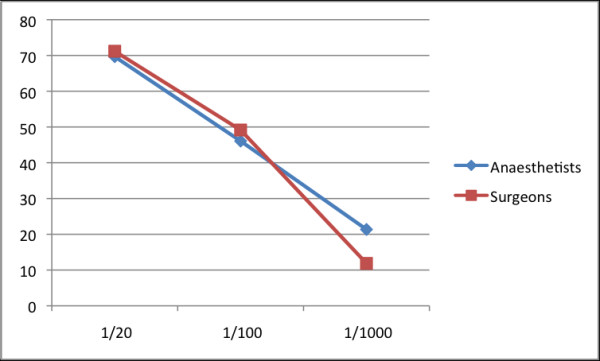
**Disclosure of minor risk based on incidence**. Percentage of anaesthetists and surgeons who agreed to statements related to disclosing minor risk dependant on incidence.

**Figure 2:**
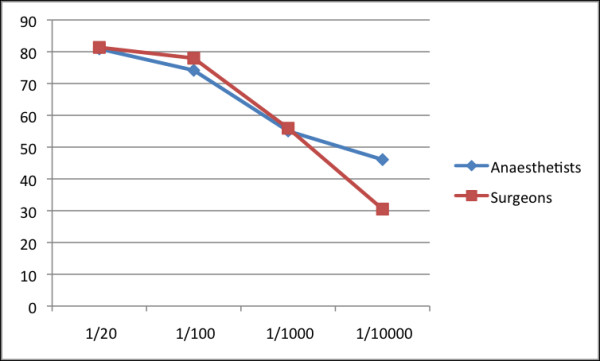
**Disclosure of major risk based on incidence**. Percentage of anaesthetists and surgeons who agreed to statements related to disclosing major risk dependant on incidence.

The level of risk disclosure that respondents felt appropriate is shown for minor risk in Figure 1 and for major risk in Figure 2. Over 50% of both surgeons and anaesthetists felt that major risks with an incidence of >1 in 1000 or more should be disclosed. However, more anaesthetists than surgeons felt that major risks of incidences of >1/10000 should be disclosed to patients as part of the consent process. Seventy percent of both anaesthetists and surgeons felt that minor risks with an incidence of >1 in 20 should be disclosed to patient when obtaining consent.

## Discussion

We believe that this is the first study of anaesthetists' and surgeons' attitudes to consent since the publication of UK guidance on this issue. We have observed that the majority of participants felt that the consent process should include an explanation of what the procedure entails and that one of the main purposes of the consent process was to respect patients' right to autonomy. Our sample of participants however had a significant minority of respondents with an apparently paternalistic attitude towards the consent process. Several anaesthetists believed that the consent process was inappropriate as information disclosed may be confusing to patients or may dissuade them from undergoing the procedure. This goes against the current guidelines and legal position that the patient should be told 'what a reasonable patient in the patient's position would want to know' [21]. There may be some evidence to support such views as a study showed that up to 40% of patients feel more anxious after being informed of the risks of their procedure [22] and there are reports that conveying rare complications will lead to an information overload of which there is no guarantee that the patient will retain or correctly understand the risk information [6,10]. Previous studies have shown that patients vary in the amount of information they want and are able to comprehend and retain [22]. This view if maintained in our group of participants as over 50% felt that patients do not usually remember all the information given to them during the consent process. More educated patients are also more actively involved in decision-making with regards to their treatment [3] and patients' IQ and age have an effect on information recall and understanding [22,23].

Our results suggest that the participants of this study strongly agree that the patient must be informed about what the procedure entails, what it aims to achieve, any additional procedure required afterwards, the realistic outcomes of treatment and the possibility of death or significant morbidity. The provision of such details is in standing with current guidelines and an essential part of the consent process. This is supported by studies reporting that preparatory information about a procedure and its risks improves patients' compliance and post-procedure progress, in addition to reducing post-operative medication use [24,25]. However, some patients may not want to know such details [26]. Dawes and colleagues [3] found that most patients were interested to know about the important complications of their surgery, with 38% wanting to know all complications and, conversely, 18% wishing not to be informed of any complications. Nearly half wanted an explanation of their treatment while 20% did not want to know what the operation involved. It may therefore not be possible for an anaesthetist or surgeon to know 'what a reasonable patient in the patient's position would want to know' [21].

Historically 1% prevalence was viewed as the threshold below which a patient should not be informed of a particular risk [10,24]. Our findings confirm that this attitude remains for a big majority of respondents. Seventy five percent of the participants agreed that only major hazards of more than 1% incidence should be disclosed, compared to 55% for risks above 1 in 1,000 and 45% for risks above 1 in 10,000. Our data suggest that anaesthetists, more than surgeons, are in favour of informing patients about major hazards occurring more commonly than 1 in 10,000, though this is in need of further study. This may reflect the nature of rare anaesthesia related complications, which while rare may have major impact such as hypoxic brain injury or permanent neurological injury. Such variation in opinions support pre-existing understanding that there is still no consensus on the fundamental question of what constitutes a significant risk that requires communication [13,23,27]. This study was undertaken before the GMC guidance on consent was published [28] but after the publication of the AAGBI consent guidelines [29] and the Department of Health (UK) guides on good practice in consent implementation [30]. The AAGBI guidance is explicit that 'rare but serious complications...should be included in written information, as should the very small risk of death.' The new GMC guidance echoes this; 'you must tell patients if an investigation or treatment might result in a serious adverse outcome, even if the likelihood is very small.'

The questionnaire used in this study was structured to elicit specific information from the participants; however, this form of questionnaire does not permit participants to express concerns that are not mentioned within its structured format, which may mean that some issues may have been overlooked. Furthermore the five-point Likert scale used in the questionnaire is vulnerable to certain forms of bias and distortion [19] such as 'central tendency bias' where respondents avoid using extreme response categories, a finding that was observed only amongst a minority of the participants and was minimized by excluding the midpoint answer (point 3) which could be argued as meaning partially agree or disagree or don't know. In addition the results may have been affected by 'social desirability bias' when respondents try to portray themselves or an organization 'in a favourable light'. The questionnaires in our study were anonymous which may have helped reduce this latter form of bias. Although our sample of anaesthetists reflects the demographic profile of the English anaesthetic and surgical workforce, there is always a question of how representative a sample is. We found no clear differences amongst surgeons or anaesthetists between the three hospitals, which are of differing sizes and in completely different geographical areas, suggesting that our results are likely to be broadly applicable. The questionnaires intra-group correlation was strong other than Table 5 (0.05) suggesting that the questions in this group were not reliable. The inter-group correlation of 0.6 suggests an adequate degree of correlation among the differing question groups.

The response rate in our study (63%) could be considered low. It is accepted that there may have been elements of reporter bias in that those interested in the informed consent process are more likely to respond, but such bias was probably diluted by the high sample size.

We feel that highlighting the opinions of anaesthetists and surgeons can help in identifying how the consent process may be improved upon. The observation that there were still respondents with potentially paternalistic attitudes among the studied population raises important questions regarding how the process of consenting patient is conducted and whether it is in standing with current national guidelines. Our findings open up the doorway for more work in improving awareness and training among anaesthetists and surgeons on how to approach the consent process. Further research into attitudes to the consent process amongst clinicians is needed both to reproduce the results from this study, and also to conduct more in depth, qualitative interviews with individual respondents.

## Conclusions

We have reported the first survey of anaesthetists' and surgeons' attitudes towards consent since the introduction of the AAGBI and Department of Health guidance. The study highlighted that the majority of the studies population agreed with statements reflecting current legal and ethical understanding regarding consent however there were a considerable minority with divergent views.

## Competing interests

The authors declare that they have no competing interests.

## Authors' contributions

AABJ: Assisted with conception and design of the study, distributed questionnaires and had a major part in preparation of the final manuscript. SW: Distributed questionnaires and had a supervising role in preparing the final manuscript. SMW: Distributed questionnaires and had a role in preparing the final manuscript. JGH: Assisted with conception and design of the study and had a supervising role in preparing the final manuscript. IKM: Assisted with conception and design of the study, distributed questionnaires and had a supervising role in preparing the final manuscript

*All authors have read and approved the final manuscript

## Pre-publication history

The pre-publication history for this paper can be accessed here:

http://www.biomedcentral.com/1472-6939/11/2/prepub

## Supplementary Material

Additional file 1 **Informed Consent Questionnaire**. Thirty-five point questionnaire assessing attitudes towards informed consent. Click here for file
