# Supplementary material for: Anaesthetists' and surgeons' attitudes towards informed consent in the UK: an observational study
Source: BMC Med Ethics. 2010 Feb 23;11:2. doi: 10.1186/1472-6939-11-2 (PMC2834584; doi:10.1186/1472-6939-11-2)
Supplement: Additional file 1 — Informed Consent Questionnaire. Thirty-five point questionnaire assessing attitudes towards informed consent. [file 1472-6939-11-2-S1.DOC]

**Questionnaire assessing doctor’s attitudes towards informed consent**

In recent years there has been a movement towards a more patient orientated approach to obtaining informed consent and doctors must meet new standards to satisfy patient autonomy while protecting themselves from medical litigation.

This questionnaire is concerned with assessing doctors’ attitudes towards informed consent.

**Details of participating doctor:**

**Age:**

| o 21 - 30 | o 31 - 40 | o 41 - 50 | o 51 - 60 | o >60 |
| --- | --- | --- | --- | --- |

**Sex:**

| o Male | o Female |
| --- | --- |

**Position:**

| o Consultant | o Registrar | o SHO |
| --- | --- | --- |

**Specialty:**

| o Surgery | o Anaesthesia |
| --- | --- |

Following are statements regarding **the process of consent**

Please **state to what extent you agree** with each statement by circling the appropriate number (1 = completely disagree - 5 = completely agree):

| **The main purposes of informed consent are to:** |  |  |  |  |  |
| --- | --- | --- | --- | --- | --- |
| Inform the patient about possible risks/complications | 1 | 2 | 3 | 4 | 5 |
| Respect the patient’s right of autonomy | 1 | 2 | 3 | 4 | 5 |
| Educate the patient about alternative treatment options | 1 | 2 | 3 | 4 | 5 |
| Provide the doctor with greater protection against medical litigation | 1 | 2 | 3 | 4 | 5 |
| Inform the patient about the desired benefits of the procedure | 1 | 2 | 3 | 4 | 5 |
| Improve the doctor patient relationship | 1 | 2 | 3 | 4 | 5 |
| Improve the patient’s compliance with medical care in general | 1 | 2 | 3 | 4 | 5 |
| Reduce patient anxiety about the procedure | 1 | 2 | 3 | 4 | 5 |
|  |  |  |  |  |  |
| **Informed consent is inappropriate/unnecessary because:** |  |  |  |  |  |
| Most patients trust their doctor to decide what is best for them | 1 | 2 | 3 | 4 | 5 |
| Most patients depend on their doctor to make the decision for them | 1 | 2 | 3 | 4 | 5 |
| Disclosing information about potentially harmful risks may be worrying and disadvantageous for the patient | 1 | 2 | 3 | 4 | 5 |
| Informing patients about details of alternative treatment modalities may be confusing | 1 | 2 | 3 | 4 | 5 |
| Discussion of risks during informed consent may dissuade the patient from undergoing a procedure that may benefit them | 1 | 2 | 3 | 4 | 5 |
| Most patients do not usually *understand* all the information given to them during the process of consent | 1 | 2 | 3 | 4 | 5 |
| Most patients do not usually *remember* all the information given to them during the process of consent | 1 | 2 | 3 | 4 | 5 |

| **During the process of informed consent the doctor should explain to the patient:** |  |  |  |  |  |
| --- | --- | --- | --- | --- | --- |
| What the procedure entails | 1 | 2 | 3 | 4 | 5 |
| What the procedure aims to achieve | 1 | 2 | 3 | 4 | 5 |
| Additional procedures that are likely to be necessary | 1 | 2 | 3 | 4 | 5 |
| A realistic outcome/results for the procedure | 1 | 2 | 3 | 4 | 5 |
| Alternative forms of the procedure | 1 | 2 | 3 | 4 | 5 |
| The possibility of death (if present) | 1 | 2 | 3 | 4 | 5 |
| The possibility of significant disability (eg: stroke / paralysis) | 1 | 2 | 3 | 4 | 5 |
| **Regarding the disclosure of *minor* risks of a procedure, information given should:** |  |  |  |  |  |
| Disclose all minor risks/complications with incidence >1/20 | 1 | 2 | 3 | 4 | 5 |
| Disclose all minor risks/complications with incidence >1/100 | 1 | 2 | 3 | 4 | 5 |
| Disclose all minor risks/complications with incidence >1/1000 | 1 | 2 | 3 | 4 | 5 |
| **Regarding the disclosure of *major* risks of a procedure, information given should:** |  |  |  |  |  |
| Disclose all major risks/complications with incidence >1/20 | 1 | 2 | 3 | 4 | 5 |
| Disclose all major risks/complications with incidence >1/100 | 1 | 2 | 3 | 4 | 5 |
| Disclose all major risks/complications with incidence >1/1000 | 1 | 2 | 3 | 4 | 5 |
| Disclose all major risks/complications with incidence >1/10000 | 1 | 2 | 3 | 4 | 5 |

Please indicate **to what degree** the following factors affect the amount of information you provide to a patient regarding anaesthesia by circling the appropriate number (1= does not affect at all / 5= strongly affects):

| Patient’s age | 1 | 2 | 3 | 4 | 5 |
| --- | --- | --- | --- | --- | --- |
| Patient’s level of education | 1 | 2 | 3 | 4 | 5 |
| Patient inquisitiveness | 1 | 2 | 3 | 4 | 5 |
| Complexity and duration of anaesthesia | 1 | 2 | 3 | 4 | 5 |
| How busy the doctor is at the time | 1 | 2 | 3 | 4 | 5 |
| Whether the patient is private or NHS | 1 | 2 | 3 | 4 | 5 |

**Thank You Aimun A Jamjoom**
